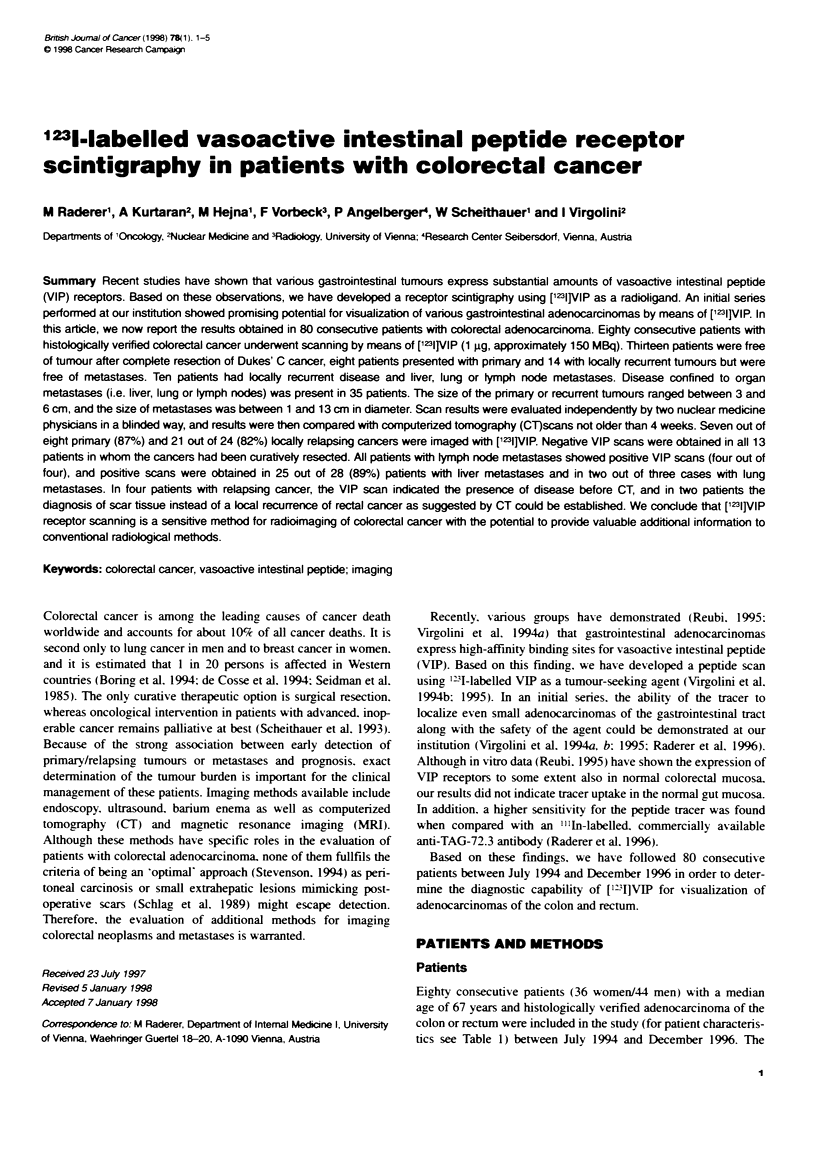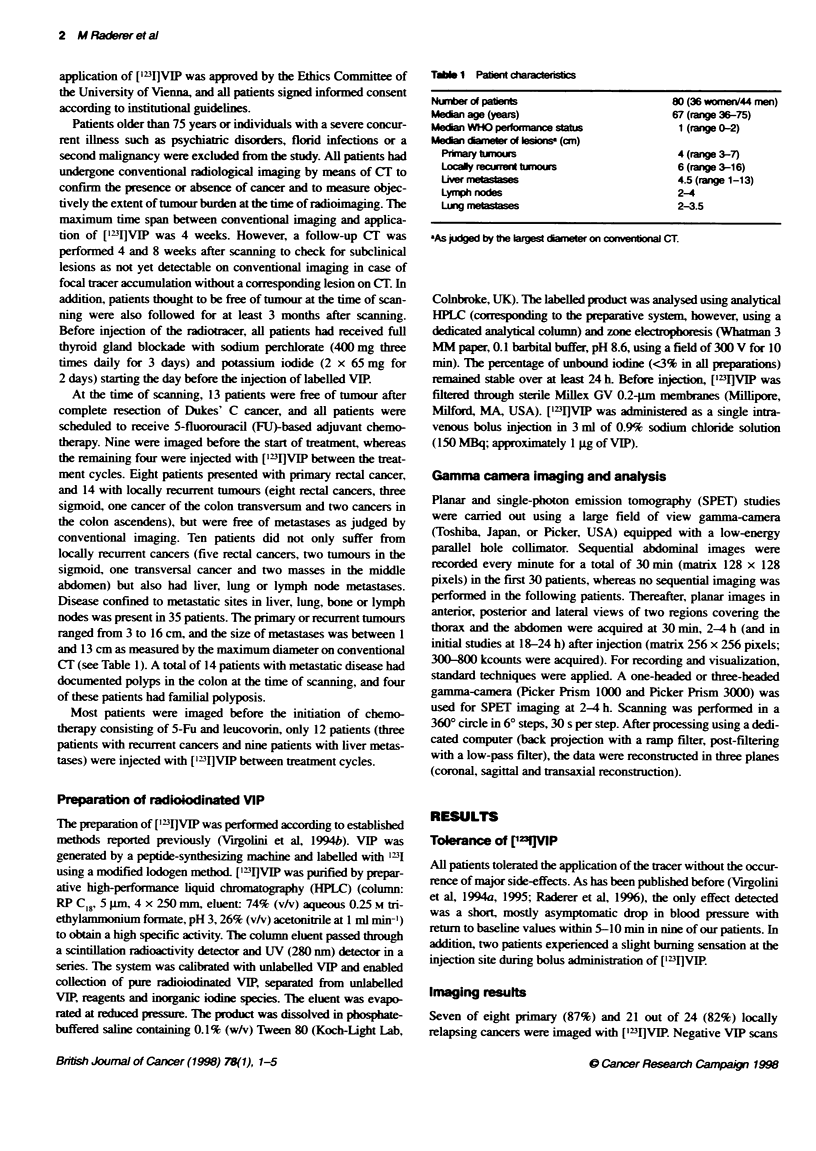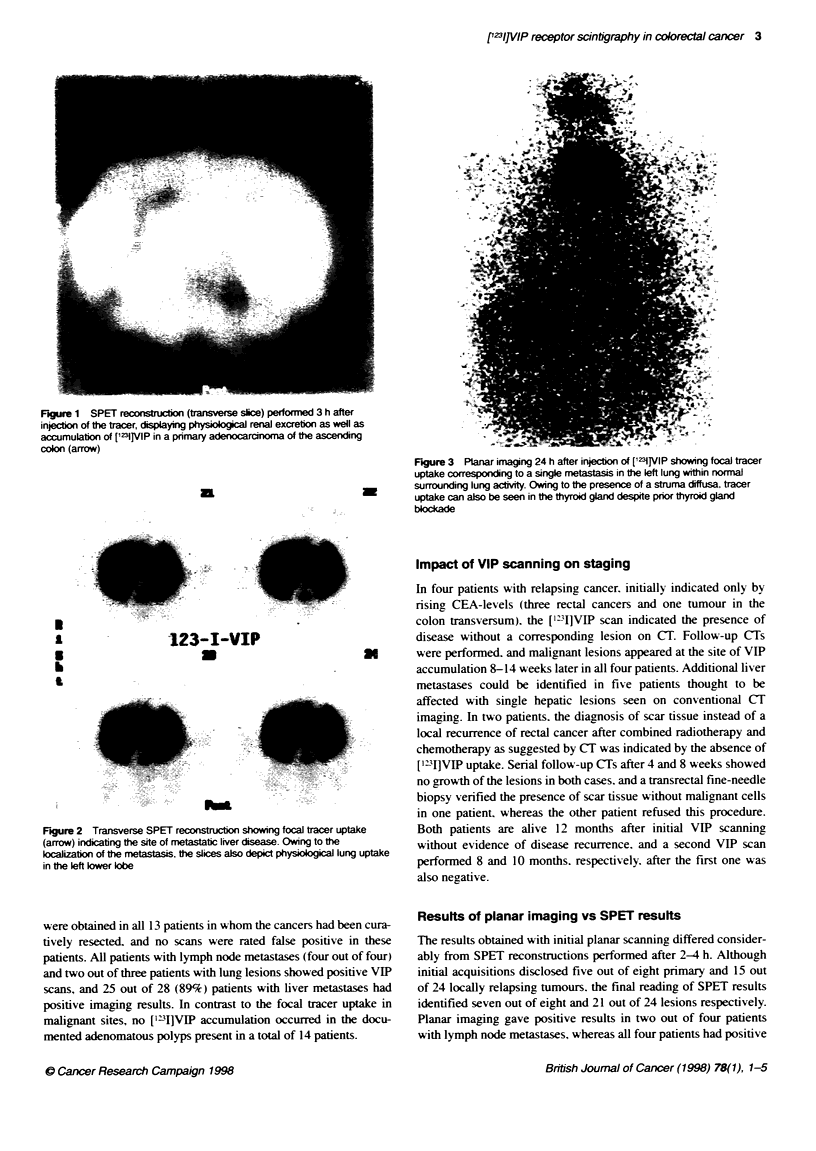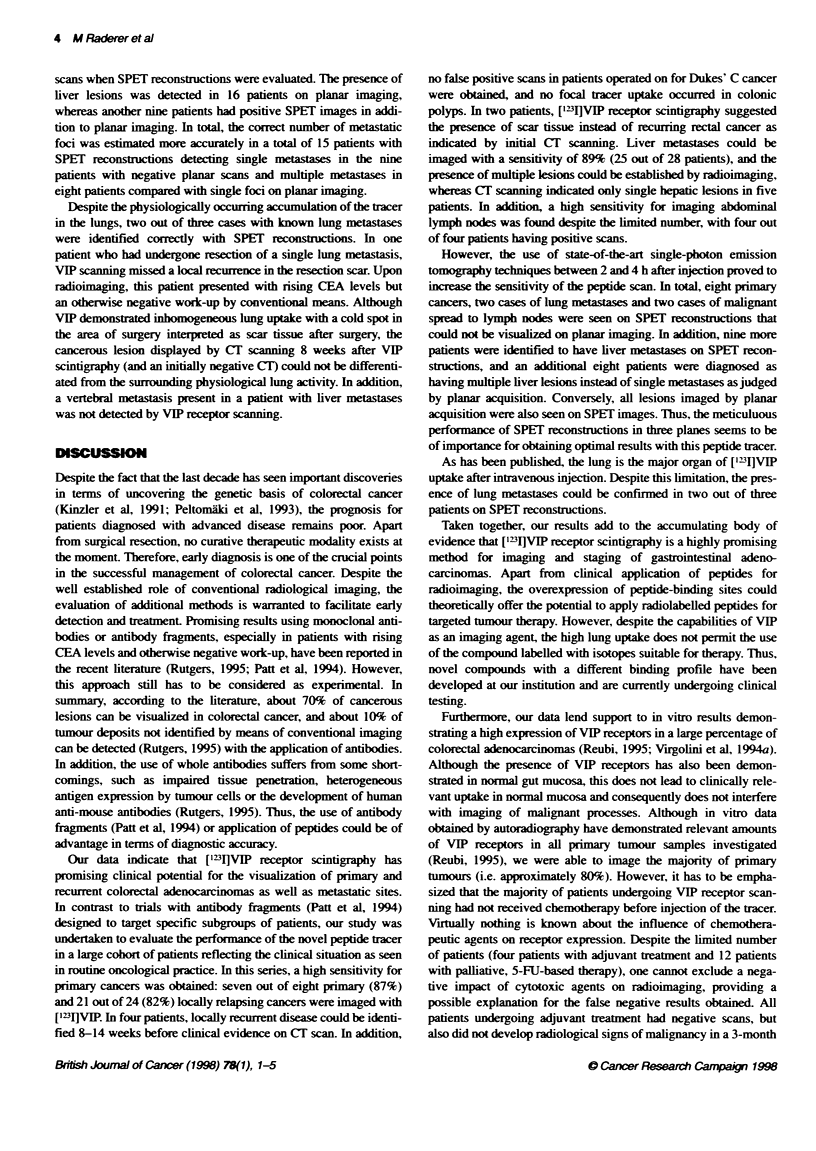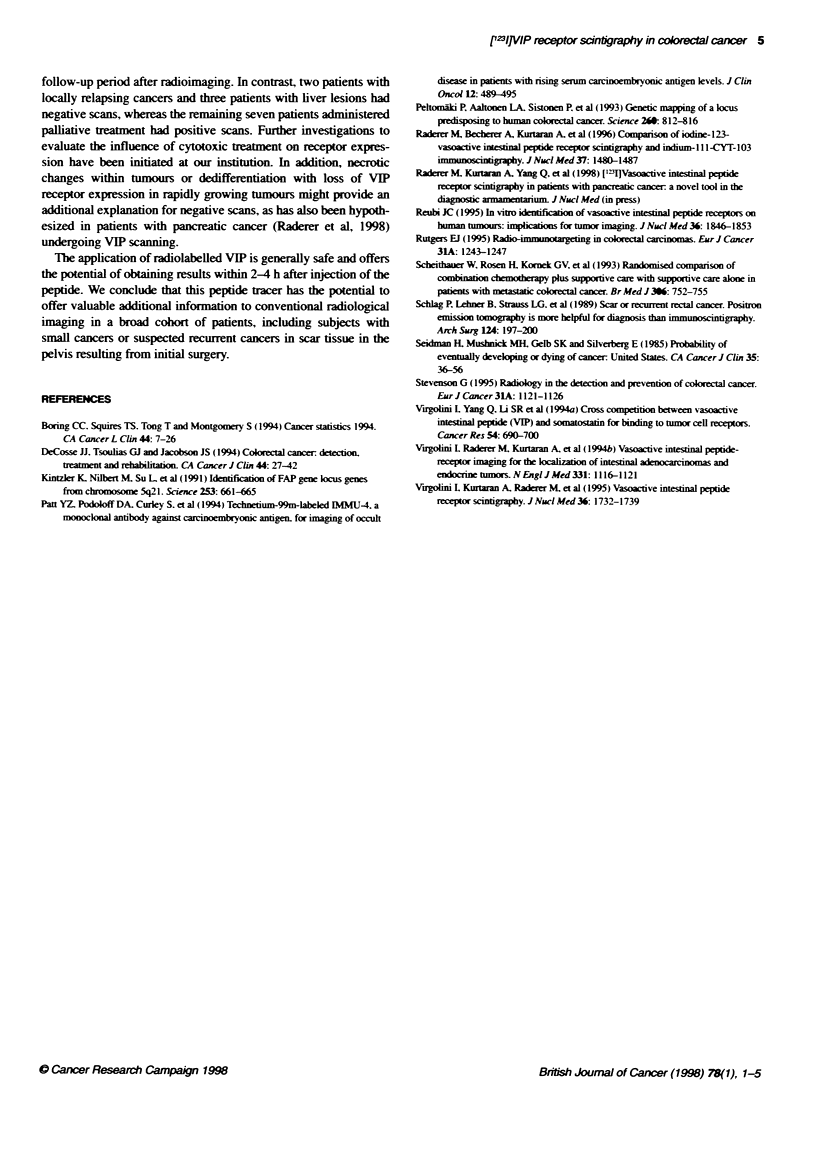# 123I-labelled vasoactive intestinal peptide receptor scintigraphy in patients with colorectal cancer.

**DOI:** 10.1038/bjc.1998.433

**Published:** 1998-07

**Authors:** M. Raderer, A. Kurtaran, M. Hejna, F. Vorbeck, P. Angelberger, W. Scheithauer, I. Virgolini

**Affiliations:** Department of Oncology, University of Vienna, Austria.

## Abstract

**Images:**


British Journal of Cancer (1998) 78(1). 1–5
© 1998 Cancer Research Campaign

### $^{123}$I-labelled vasoactive intestinal peptide receptor scintigraphy in patients with colorectal cancer

**M Raderer[1], A Kurtaran[2], M Hejna[1], F Vorbeck[3], P Angelberger[4], W Scheithauer[1] and I Virgolini[2]**

Departments of [1]Oncology, [2]Nuclear Medicine and [3]Radiology, University of Vienna; [4]Research Center Seibersdorf, Vienna, Austria

**Summary** Recent studies have shown that various gastrointestinal tumours express substantial amounts of vasoactive intestinal peptide (VIP) receptors. Based on these observations, we have developed a receptor scintigraphy using [$^{123}$I]VIP as a radioligand. An initial series performed at our institution showed promising potential for visualization of various gastrointestinal adenocarcinomas by means of [$^{123}$I]VIP. In this article, we now report the results obtained in 80 consecutive patients with colorectal adenocarcinoma. Eighty consecutive patients with histologically verified colorectal cancer underwent scanning by means of [$^{123}$I]VIP (1 µg, approximately 150 MBq). Thirteen patients were free of tumour after complete resection of Dukes' C cancer, eight patients presented with primary and 14 with locally recurrent tumours but were free of metastases. Ten patients had locally recurrent disease and liver, lung or lymph node metastases. Disease confined to organ metastases (i.e. liver, lung or lymph nodes) was present in 35 patients. The size of the primary or recurrent tumours ranged between 3 and 6 cm, and the size of metastases was between 1 and 13 cm in diameter. Scan results were evaluated independently by two nuclear medicine physicians in a blinded way, and results were then compared with computerized tomography (CT)scans not older than 4 weeks. Seven out of eight primary (87%) and 21 out of 24 (82%) locally relapsing cancers were imaged with [$^{123}$I]VIP. Negative VIP scans were obtained in all 13 patients in whom the cancers had been curatively resected. All patients with lymph node metastases showed positive VIP scans (four out of four), and positive scans were obtained in 25 out of 28 (89%) patients with liver metastases and in two out of three cases with lung metastases. In four patients with relapsing cancer, the VIP scan indicated the presence of disease before CT, and in two patients the diagnosis of scar tissue instead of a local recurrence of rectal cancer as suggested by CT could be established. We conclude that [$^{123}$I]VIP receptor scanning is a sensitive method for radioimaging of colorectal cancer with the potential to provide valuable additional information to conventional radiological methods.

**Keywords:** colorectal cancer, vasoactive intestinal peptide; imaging

Colorectal cancer is among the leading causes of cancer death worldwide and accounts for about 10% of all cancer deaths. It is second only to lung cancer in men and to breast cancer in women, and it is estimated that 1 in 20 persons is affected in Western countries (Boring et al. 1994; de Cosse et al. 1994; Seidman et al. 1985). The only curative therapeutic option is surgical resection, whereas oncological intervention in patients with advanced, inoperable cancer remains palliative at best (Scheithauer et al. 1993). Because of the strong association between early detection of primary/relapsing tumours or metastases and prognosis, exact determination of the tumour burden is important for the clinical management of these patients. Imaging methods available include endoscopy, ultrasound, barium enema as well as computerized tomography (CT) and magnetic resonance imaging (MRI). Although these methods have specific roles in the evaluation of patients with colorectal adenocarcinoma, none of them fullfils the criteria of being an 'optimal' approach (Stevenson, 1994) as peritoneal carcinosis or small extrahepatic lesions mimicking post-operative scars (Schlag et al. 1989) might escape detection. Therefore, the evaluation of additional methods for imaging colorectal neoplasms and metastases is warranted.

*Received 23 July 1997*
*Revised 5 January 1998*
*Accepted 7 January 1998*

*Correspondence to:* M Raderer, Department of Internal Medicine I, University of Vienna, Waehringer Guertel 18–20, A-1090 Vienna, Austria

Recently, various groups have demonstrated (Reubi, 1995; Virgolini et al. 1994*a*) that gastrointestinal adenocarcinomas express high-affinity binding sites for vasoactive intestinal peptide (VIP). Based on this finding, we have developed a peptide scan using $^{123}$I-labelled VIP as a tumour-seeking agent (Virgolini et al. 1994b; 1995). In an initial series, the ability of the tracer to localize even small adenocarcinomas of the gastrointestinal tract along with the safety of the agent could be demonstrated at our institution (Virgolini et al. 1994*a*, *b*; 1995; Raderer et al. 1996). Although in vitro data (Reubi, 1995) have shown the expression of VIP receptors to some extent also in normal colorectal mucosa, our results did not indicate tracer uptake in the normal gut mucosa. In addition, a higher sensitivity for the peptide tracer was found when compared with an $^{111}$In-labelled, commercially available anti-TAG-72.3 antibody (Raderer et al. 1996).

Based on these findings, we have followed 80 consecutive patients between July 1994 and December 1996 in order to determine the diagnostic capability of [$^{123}$I]VIP for visualization of adenocarcinomas of the colon and rectum.

### PATIENTS AND METHODS

### Patients

Eighty consecutive patients (36 women/44 men) with a median age of 67 years and histologically verified adenocarcinoma of the colon or rectum were included in the study (for patient characteristics see Table 1) between July 1994 and December 1996. The application of [$^{123}$I]VIP was approved by the Ethics Committee of the University of Vienna, and all patients signed informed consent according to institutional guidelines.

Patients older than 75 years or individuals with a severe concurrent illness such as psychiatric disorders, florid infections or a second malignancy were excluded from the study. All patients had undergone conventional radiological imaging by means of CT to confirm the presence or absence of cancer and to measure objectively the extent of tumour burden at the time of radioimaging. The maximum time span between conventional imaging and application of [$^{123}$I]VIP was 4 weeks. However, a follow-up CT was performed 4 and 8 weeks after scanning to check for subclinical lesions as not yet detectable on conventional imaging in case of focal tracer accumulation without a corresponding lesion on CT. In addition, patients thought to be free of tumour at the time of scanning were also followed for at least 3 months after scanning. Before injection of the radiotracer, all patients had received full thyroid gland blockade with sodium perchlorate (400 mg three times daily for 3 days) and potassium iodide (2 × 65 mg for 2 days) starting the day before the injection of labelled VIP.

At the time of scanning, 13 patients were free of tumour after complete resection of Dukes' C cancer, and all patients were scheduled to receive 5-fluorouracil (FU)-based adjuvant chemotherapy. Nine were imaged before the start of treatment, whereas the remaining four were injected with [$^{123}$I]VIP between the treatment cycles. Eight patients presented with primary rectal cancer, and 14 with locally recurrent tumours (eight rectal cancers, three sigmoid, one cancer of the colon transversum and two cancers in the colon ascendens), but were free of metastases as judged by conventional imaging. Ten patients did not only suffer from locally recurrent cancers (five rectal cancers, two tumours in the sigmoid, one transversal cancer and two masses in the middle abdomen) but also had liver, lung or lymph node metastases. Disease confined to metastatic sites in liver, lung, bone or lymph nodes was present in 35 patients. The primary or recurrent tumours ranged from 3 to 16 cm, and the size of metastases was between 1 and 13 cm as measured by the maximum diameter on conventional CT (see Table 1). A total of 14 patients with metastatic disease had documented polyps in the colon at the time of scanning, and four of these patients had familial polyposis.

Most patients were imaged before the initiation of chemotherapy consisting of 5-Fu and leucovorin, only 12 patients (three patients with recurrent cancers and nine patients with liver metastases) were injected with [$^{123}$I]VIP between treatment cycles.

### Preparation of radioiodinated VIP

The preparation of [$^{123}$I]VIP was performed according to established methods reported previously (Virgolini et al, 1994*b*). VIP was generated by a peptide-synthesizing machine and labelled with $^{123}$I using a modified Iodogen method. [$^{123}$I]VIP was purified by preparative high-performance liquid chromatography (HPLC) (column: RP $C_{18}$, 5 µm, 4 × 250 mm, eluent: 74% (v/v) aqueous 0.25 M triethylammonium formate, pH 3, 26% (v/v) acetonitrile at 1 ml min$^{-1}$) to obtain a high specific activity. The column eluent passed through a scintillation radioactivity detector and UV (280 nm) detector in a series. The system was calibrated with unlabelled VIP and enabled collection of pure radioiodinated VIP, separated from unlabelled VIP, reagents and inorganic iodine species. The eluent was evaporated at reduced pressure. The product was dissolved in phosphate-buffered saline containing 0.1% (w/v) Tween 80 (Koch-Light Lab, Colnbroke, UK). The labelled product was analysed using analytical HPLC (corresponding to the preparative system, however, using a dedicated analytical column) and zone electrophoresis (Whatman 3 MM paper, 0.1 barbital buffer, pH 8.6, using a field of 300 V for 10 min). The percentage of unbound iodine (<3% in all preparations) remained stable over at least 24 h. Before injection, [$^{123}$I]VIP was filtered through sterile Millex GV 0.2-µm membranes (Millipore, Milford, MA, USA). [$^{123}$I]VIP was administered as a single intravenous bolus injection in 3 ml of 0.9% sodium chloride solution (150 MBq; approximately 1 µg of VIP).

**Table 1** Patient characteristics

| | |
|---|---|
| Number of patients | 80 (36 women/44 men) |
| Median age (years) | 67 (range 36–75) |
| Median WHO performance status | 1 (range 0–2) |
| Median diameter of lesions[a] (cm) | |
| Primary tumours | 4 (range 3–7) |
| Locally recurrent tumours | 6 (range 3–16) |
| Liver metastases | 4.5 (range 1–13) |
| Lymph nodes | 2–4 |
| Lung metastases | 2–3.5 |

[a]As judged by the largest diameter on conventional CT.

### Gamma camera imaging and analysis

Planar and single-photon emission tomography (SPET) studies were carried out using a large field of view gamma-camera (Toshiba, Japan, or Picker, USA) equipped with a low-energy parallel hole collimator. Sequential abdominal images were recorded every minute for a total of 30 min (matrix 128 × 128 pixels) in the first 30 patients, whereas no sequential imaging was performed in the following patients. Thereafter, planar images in anterior, posterior and lateral views of two regions covering the thorax and the abdomen were acquired at 30 min, 2–4 h (and in initial studies at 18–24 h) after injection (matrix 256 × 256 pixels; 300–800 kcounts were acquired). For recording and visualization, standard techniques were applied. A one-headed or three-headed gamma-camera (Picker Prism 1000 and Picker Prism 3000) was used for SPET imaging at 2–4 h. Scanning was performed in a 360° circle in 6° steps, 30 s per step. After processing using a dedicated computer (back projection with a ramp filter, post-filtering with a low-pass filter), the data were reconstructed in three planes (coronal, sagittal and transaxial reconstruction).

### RESULTS

### Tolerance of [$^{123}$I]VIP

All patients tolerated the application of the tracer without the occurrence of major side-effects. As has been published before (Virgolini et al, 1994*a*, 1995; Raderer et al, 1996), the only effect detected was a short, mostly asymptomatic drop in blood pressure with return to baseline values within 5–10 min in nine of our patients. In addition, two patients experienced a slight burning sensation at the injection site during bolus administration of [$^{123}$I]VIP.

### Imaging results

Seven of eight primary (87%) and 21 out of 24 (82%) locally relapsing cancers were imaged with [$^{123}$I]VIP. Negative VIP scans

© Cancer Research Campaign 1998

**Figure 1** SPET reconstruction (transverse slice) performed 3 h after injection of the tracer, displaying physiological renal excretion as well as accumulation of [$^{123}$I]VIP in a primary adenocarcinoma of the ascending colon (arrow)

**Figure 2** Transverse SPET reconstruction showing focal tracer uptake (arrow) indicating the site of metastatic liver disease. Owing to the localization of the metastasis, the slices also depict physiological lung uptake in the left lower lobe were obtained in all 13 patients in whom the cancers had been curatively resected, and no scans were rated false positive in these patients. All patients with lymph node metastases (four out of four) and two out of three patients with lung lesions showed positive VIP scans, and 25 out of 28 (89%) patients with liver metastases had positive imaging results. In contrast to the focal tracer uptake in malignant sites, no [$^{123}$I]VIP accumulation occurred in the documented adenomatous polyps present in a total of 14 patients.

**Figure 3** Planar imaging 24 h after injection of [$^{123}$I]VIP showing focal tracer uptake corresponding to a single metastasis in the left lung within normal surrounding lung activity. Owing to the presence of a struma diffusa, tracer uptake can also be seen in the thyroid gland despite prior thyroid gland blockade

### Impact of VIP scanning on staging

In four patients with relapsing cancer, initially indicated only by rising CEA-levels (three rectal cancers and one tumour in the colon transversum), the [$^{123}$I]VIP scan indicated the presence of disease without a corresponding lesion on CT. Follow-up CTs were performed, and malignant lesions appeared at the site of VIP accumulation 8–14 weeks later in all four patients. Additional liver metastases could be identified in five patients thought to be affected with single hepatic lesions seen on conventional CT imaging. In two patients, the diagnosis of scar tissue instead of a local recurrence of rectal cancer after combined radiotherapy and chemotherapy as suggested by CT was indicated by the absence of [$^{123}$I]VIP uptake. Serial follow-up CTs after 4 and 8 weeks showed no growth of the lesions in both cases, and a transrectal fine-needle biopsy verified the presence of scar tissue without malignant cells in one patient, whereas the other patient refused this procedure. Both patients are alive 12 months after initial VIP scanning without evidence of disease recurrence, and a second VIP scan performed 8 and 10 months, respectively, after the first one was also negative.

### Results of planar imaging vs SPET results

The results obtained with initial planar scanning differed considerably from SPET reconstructions performed after 2–4 h. Although initial acquisitions disclosed five out of eight primary and 15 out of 24 locally relapsing tumours, the final reading of SPET results identified seven out of eight and 21 out of 24 lesions respectively. Planar imaging gave positive results in two out of four patients with lymph node metastases, whereas all four patients had positive

© Cancer Research Campaign 1998

scans when SPET reconstructions were evaluated. The presence of liver lesions was detected in 16 patients on planar imaging, whereas another nine patients had positive SPET images in addition to planar imaging. In total, the correct number of metastatic foci was estimated more accurately in a total of 15 patients with SPET reconstructions detecting single metastases in the nine patients with negative planar scans and multiple metastases in eight patients compared with single foci on planar imaging.

Despite the physiologically occurring accumulation of the tracer in the lungs, two out of three cases with known lung metastases were identified correctly with SPET reconstructions. In one patient who had undergone resection of a single lung metastasis, VIP scanning missed a local recurrence in the resection scar. Upon radioimaging, this patient presented with rising CEA levels but an otherwise negative work-up by conventional means. Although VIP demonstrated inhomogeneous lung uptake with a cold spot in the area of surgery interpreted as scar tissue after surgery, the cancerous lesion displayed by CT scanning 8 weeks after VIP scintigraphy (and an initially negative CT) could not be differentiated from the surrounding physiological lung activity. In addition, a vertebral metastasis present in a patient with liver metastases was not detected by VIP receptor scanning.

### DISCUSSION

Despite the fact that the last decade has seen important discoveries in terms of uncovering the genetic basis of colorectal cancer (Kinzler et al, 1991; Peltomäki et al, 1993), the prognosis for patients diagnosed with advanced disease remains poor. Apart from surgical resection, no curative therapeutic modality exists at the moment. Therefore, early diagnosis is one of the crucial points in the successful management of colorectal cancer. Despite the well established role of conventional radiological imaging, the evaluation of additional methods is warranted to facilitate early detection and treatment. Promising results using monoclonal antibodies or antibody fragments, especially in patients with rising CEA levels and otherwise negative work-up, have been reported in the recent literature (Rutgers, 1995; Patt et al, 1994). However, this approach still has to be considered as experimental. In summary, according to the literature, about 70% of cancerous lesions can be visualized in colorectal cancer, and about 10% of tumour deposits not identified by means of conventional imaging can be detected (Rutgers, 1995) with the application of antibodies. In addition, the use of whole antibodies suffers from some shortcomings, such as impaired tissue penetration, heterogeneous antigen expression by tumour cells or the development of human anti-mouse antibodies (Rutgers, 1995). Thus, the use of antibody fragments (Patt et al, 1994) or application of peptides could be of advantage in terms of diagnostic accuracy.

Our data indicate that $[^{123}I]$VIP receptor scintigraphy has promising clinical potential for the visualization of primary and recurrent colorectal adenocarcinomas as well as metastatic sites. In contrast to trials with antibody fragments (Patt et al, 1994) designed to target specific subgroups of patients, our study was undertaken to evaluate the performance of the novel peptide tracer in a large cohort of patients reflecting the clinical situation as seen in routine oncological practice. In this series, a high sensitivity for primary cancers was obtained: seven out of eight primary (87%) and 21 out of 24 (82%) locally relapsing cancers were imaged with $[^{123}I]$VIP. In four patients, locally recurrent disease could be identified 8–14 weeks before clinical evidence on CT scan. In addition, no false positive scans in patients operated on for Dukes' C cancer were obtained, and no focal tracer uptake occurred in colonic polyps. In two patients, $[^{123}I]$VIP receptor scintigraphy suggested the presence of scar tissue instead of recurring rectal cancer as indicated by initial CT scanning. Liver metastases could be imaged with a sensitivity of 89% (25 out of 28 patients), and the presence of multiple lesions could be established by radioimaging, whereas CT scanning indicated only single hepatic lesions in five patients. In addition, a high sensitivity for imaging abdominal lymph nodes was found despite the limited number, with four out of four patients having positive scans.

However, the use of state-of-the-art single-photon emission tomography techniques between 2 and 4 h after injection proved to increase the sensitivity of the peptide scan. In total, eight primary cancers, two cases of lung metastases and two cases of malignant spread to lymph nodes were seen on SPET reconstructions that could not be visualized on planar imaging. In addition, nine more patients were identified to have liver metastases on SPET reconstructions, and an additional eight patients were diagnosed as having multiple liver lesions instead of single metastases as judged by planar acquisition. Conversely, all lesions imaged by planar acquisition were also seen on SPET images. Thus, the meticulous performance of SPET reconstructions in three planes seems to be of importance for obtaining optimal results with this peptide tracer.

As has been published, the lung is the major organ of $[^{123}I]$VIP uptake after intravenous injection. Despite this limitation, the presence of lung metastases could be confirmed in two out of three patients on SPET reconstructions.

Taken together, our results add to the accumulating body of evidence that $[^{123}I]$VIP receptor scintigraphy is a highly promising method for imaging and staging of gastrointestinal adenocarcinomas. Apart from clinical application of peptides for radioimaging, the overexpression of peptide-binding sites could theoretically offer the potential to apply radiolabelled peptides for targeted tumour therapy. However, despite the capabilities of VIP as an imaging agent, the high lung uptake does not permit the use of the compound labelled with isotopes suitable for therapy. Thus, novel compounds with a different binding profile have been developed at our institution and are currently undergoing clinical testing.

Furthermore, our data lend support to in vitro results demonstrating a high expression of VIP receptors in a large percentage of colorectal adenocarcinomas (Reubi, 1995; Virgolini et al, 1994*a*). Although the presence of VIP receptors has also been demonstrated in normal gut mucosa, this does not lead to clinically relevant uptake in normal mucosa and consequently does not interfere with imaging of malignant processes. Although in vitro data obtained by autoradiography have demonstrated relevant amounts of VIP receptors in all primary tumour samples investigated (Reubi, 1995), we were able to image the majority of primary tumours (i.e. approximately 80%). However, it has to be emphasized that the majority of patients undergoing VIP receptor scanning had not received chemotherapy before injection of the tracer. Virtually nothing is known about the influence of chemotherapeutic agents on receptor expression. Despite the limited number of patients (four patients with adjuvant treatment and 12 patients with palliative, 5-FU-based therapy), one cannot exclude a negative impact of cytotoxic agents on radioimaging, providing a possible explanation for the false negative results obtained. All patients undergoing adjuvant treatment had negative scans, but also did not develop radiological signs of malignancy in a 3-month

© Cancer Research Campaign 1998

follow-up period after radioimaging. In contrast, two patients with locally relapsing cancers and three patients with liver lesions had negative scans, whereas the remaining seven patients administered palliative treatment had positive scans. Further investigations to evaluate the influence of cytotoxic treatment on receptor expression have been initiated at our institution. In addition, necrotic changes within tumours or dedifferentiation with loss of VIP receptor expression in rapidly growing tumours might provide an additional explanation for negative scans, as has also been hypothesized in patients with pancreatic cancer (Raderer et al, 1998) undergoing VIP scanning.

The application of radiolabelled VIP is generally safe and offers the potential of obtaining results within 2–4 h after injection of the peptide. We conclude that this peptide tracer has the potential to offer valuable additional information to conventional radiological imaging in a broad cohort of patients, including subjects with small cancers or suspected recurrent cancers in scar tissue in the pelvis resulting from initial surgery.

### REFERENCES

Boring CC, Squires TS, Tong T and Montgomery S (1994) Cancer statistics 1994. *CA Cancer L Clin* **44**: 7–26

DeCosse JJ, Tsoulias GJ and Jacobson JS (1994) Colorectal cancer: detection, treatment and rehabilitation. *CA Cancer J Clin* **44**: 27–42

Kintzler K, Nilbert M, Su L, et al (1991) Identification of FAP gene locus genes from chromosome 5q21. *Science* **253**: 661–665

Patt YZ, Podoloff DA, Curley S, et al (1994) Technetium-99m-labeled IMMU-4, a monoclonal antibody against carcinoembryonic antigen, for imaging of occult disease in patients with rising serum carcinoembryonic antigen levels. *J Clin Oncol* **12**: 489–495

Peltomäki P, Aaltonen LA, Sistonen P, et al (1993) Genetic mapping of a locus predisposing to human colorectal cancer. *Science* **260**: 812–816

Raderer M, Becherer A, Kurtaran A, et al (1996) Comparison of iodine-123-vasoactive intestinal peptide receptor scintigraphy and indium-111-CYT-103 immunoscintigraphy. *J Nucl Med* **37**: 1480–1487

Raderer M, Kurtaran A, Yang Q, et al (1998) [$^{123}$I]Vasoactive intestinal peptide receptor scintigraphy in patients with pancreatic cancer: a novel tool in the diagnostic armamentarium. *J Nucl Med* (in press)

Reubi JC (1995) In vitro identification of vasoactive intestinal peptide receptors on human tumours: implications for tumor imaging. *J Nucl Med* **36**: 1846–1853

Rutgers EJ (1995) Radio-immunotargeting in colorectal carcinomas. *Eur J Cancer* **31A**: 1243–1247

Scheithauer W, Rosen H, Kornek GV, et al (1993) Randomised comparison of combination chemotherapy plus supportive care with supportive care alone in patients with metastatic colorectal cancer. *Br Med J* **306**: 752–755

Schlag P, Lehner B, Strauss LG, et al (1989) Scar or recurrent rectal cancer. Positron emission tomography is more helpful for diagnosis than immunoscintigraphy. *Arch Surg* **124**: 197–200

Seidman H, Mushnick MH, Gelb SK and Silverberg E (1985) Probability of eventually developing or dying of cancer: United States. *CA Cancer J Clin* **35**: 36–56

Stevenson G (1995) Radiology in the detection and prevention of colorectal cancer. *Eur J Cancer* **31A**: 1121–1126

Virgolini I, Yang Q, Li SR et al (1994*a*) Cross competition between vasoactive intestinal peptide (VIP) and somatostatin for binding to tumor cell receptors. *Cancer Res* **54**: 690–700

Virgolini I, Raderer M, Kurtaran A, et al (1994*b*) Vasoactive intestinal peptide-receptor imaging for the localization of intestinal adenocarcinomas and endocrine tumors. *N Engl J Med* **331**: 1116–1121

Virgolini I, Kurtaran A, Raderer M, et al (1995) Vasoactive intestinal peptide receptor scintigraphy. *J Nucl Med* **36**: 1732–1739

© Cancer Research Campaign 1998